# Evaluating the Predictive Value of Frontal White Matter Hyperintensity Burden on Cognitive Response to NAC and Exercise Therapy in Vascular Mild Cognitive Impairment: Findings from the MOVE‐IT Trial

**DOI:** 10.1002/alz70856_105730

**Published:** 2026-01-08

**Authors:** Ethan Mah, Damien Gallagher, Nathan Herrmann, Sandra E. Black, Joel Ramirez, Kate Survilla, Danielle Vieira, Jinghan Jenny Chen, Yejin Kang, Paul I. Oh, Susan Marzolini, Ana C. Andreazza, Alex Kiss, Walter Swardfager, Krista L Lanctôt

**Affiliations:** ^1^ University of Toronto, Toronto, ON, Canada; ^2^ Neuropsychopharmacology Research Group, Sunnybrook Research Institute, Toronto, ON, Canada; ^3^ Department of Psychiatry, Temerty Faculty of Medicine, University of Toronto, Toronto, ON, Canada; ^4^ Geriatric Psychopharmacology Research Group, Sunnybrook Research Institute, Toronto, ON, Canada; ^5^ Dr. Sandra E. Black Centre for Brain Resilience and Recovery, LC Campbell Cognitive Neurology, Hurvitz Brain Sciences Program, Sunnybrook Research Institute, University of Toronto, Toronto, ON, Canada; ^6^ KITE Research Institute, University Health Network, Toronto, ON, Canada; ^7^ Hurvitz Brain Sciences Program, Sunnybrook Research Institute, Toronto, ON, Canada

## Abstract

**Background:**

White matter hyperintensities (WMHs) in persons with vascular mild cognitive impairment (vMCI), particularly frontal WMHs, have been associated with decreased executive function (EF). While N‐acetylcysteine (NAC) and exercise therapy may improve EF in patients with vMCI, identifying predictors of response could help personalize treatment. We hypothesized that lower baseline frontal WMH volume in vMCI patients treated with NAC and exercise will correlate with increasing improvement in EF over 6 months of treatment.

**Method:**

Participants received 24 weeks of NAC or placebo in combination with exercise therapy (*n* = 60) as part of the Efficacy and Safety of N‐acetylcysteine in patients with mild vascular cognitive impairment (MOVE‐IT) clinical trial. Composite z scores for EF were calculated using Trail Making Part Test B, F‐A‐S Test, and Animal Naming Test administered at baseline, week 12, and week 24. Frontal WMH volumes were captured from baseline MRI scans. Linear mixed models were used that accounted for sex differences, age, years of education, and baseline executive function.

**Result:**

Both treatment groups had a significant improvement in EF at midpoint (β = 0.194, SE = 0.059, *p* =  0.002) and endpoint (β = 0.342, SE = 0.058, *p* < 0.001). Lower baseline frontal WMH and treatment group was not associated with greater improvement in EF at midpoint (β = ‐0.260, SE = 0.144, *p* =  0.075) or at endpoint (β = ‐0.162, SE = 0.144, *p* =  0.266). Lower baseline frontal WMH volume was significantly associated with greater improvement in performance on the Trail Making Test Part B at midpoint (β = ‐0.597, SE = 0.197, *p* =  0.003) but not at endpoint (β = ‐0.328, SE = 0.197, *p* =  0.099) among participants randomized to the NAC and exercise group, but not the placebo and exercise group.

**Conclusion:**

This study found a predictive value of frontal WMH burden for novel interventions aiming to improve cognitive outcomes in patients with vMCI. WMH burden could help guide the development of targeted, effective therapies that address the complexities of cognitive impairment in aging populations.

